# An atrial septal aneurysm with an organized thrombus in an asymptomatic patient: A case report

**DOI:** 10.1097/MD.0000000000018074

**Published:** 2019-11-27

**Authors:** Xinxin Wang, Chunguo Wang, Dehua Ma, Jiang Jin, Bo Zhang, Chengchu Zhu, Baofu Chen

**Affiliations:** Department of Thoracic and Cardiovascular Surgery, Affiliated Taizhou Hospital of Wenzhou Medical University, Taizhou, China.

**Keywords:** atrial septal aneurysm, atrial septal defect, case report, thrombus

## Abstract

**Introduction:**

An atrial septal aneurysm (ASA) is a rare congenital cardiac deformity characterized by interatrial septum protruding into atria forming a saccular structure.

**Patient concerns:**

In our case, a 42-year-old female patient presented to our hospital complained of palpitation.

**Diagnosis:**

Transthoracic echocardiography detected a 3.4 × 3.4 cm circular mass attached to the interatrial septum in right atrium complicated with a 6 mm secundum atrial septal defects (ASD).

**Interventions:**

The patient received a cardiopulmonary bypass surgery to remove the mass and close the ASD.

**Outcomes:**

The mass turned out to be an organized thrombus with calcium deposition and fibrinoid necrosis.

**Conclusion:**

ASA is a potential location of atrial thrombus because of the stagnation of blood. Systemic embolism events are the main complications of ASA. Surgery or anticoagulation is both recommended in patients with ASA with thrombus.

## Abstract 2

Supplemental Digital Content is available in the text

## 1 Introduction

An atrial septal aneurysm (ASA) is a “saccular” interatrial septal tissue located at the level of the fossa ovalis, which protrudes to the right or the left atrium or both for more than 15 mm beyond the plane of the atrial septum during the cardiorespiratory cycle.^[1]^ ASA is associated with pulmonary and systemic embolism. It could be detected noninvasively on transesophageal echocardiography (TEE) with a prevalence of 2% to 10%.^[2]^ ASA complicated with embolism events had been reported. Here we present a rare case of ASA with organized thrombus but without any embolism.

## 2 Case presentation

**Figure 1:**
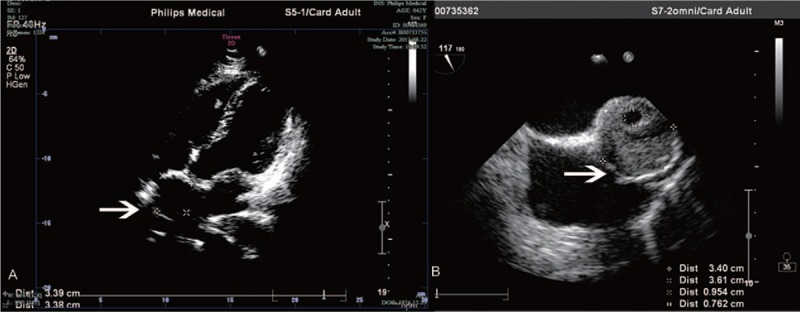
The echocardiography shows a mass in the right atrium. (A) Transthoracic echocardiography and (B) transesophageal echocardiography.

**Figure 2:**
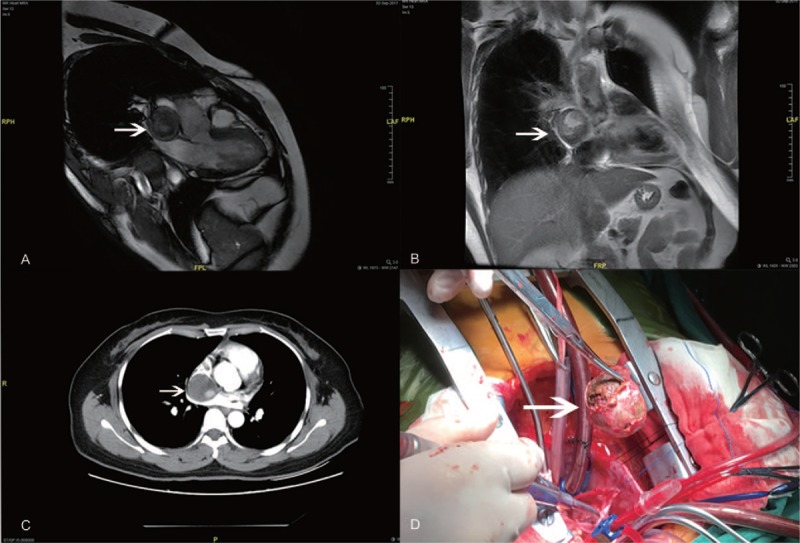
The magnetic resonance imaging and contrast enhanced computed tomography shows a heterogeneous mass without enhancement (A, B, and C). The mass was peeled thoroughly from the septum during operation (D).

A 42-year-old woman presented to our hospital with palpitation for 30 days. She had no history of cerebrovascular accidents or cardiomyopathy. Physical examination was unremarkable. A routine 12-lead electrocardiogram (ECG) revealed sinus rhythm and atrial premature beats. A further 24-hours holter recorded with sinus arrhythmia, atrial premature beats, paroxymal atrial tachycardia, and incomplete right bundle branch block (IBBB). Transthoracic echocardiography (TTE) was recommended first and detected a circular mass of 3.4 × 3.4 cm attached to the interatrial septum protruding to the right atrium. The mass was heterogeneously echogenic and not pedunculated (Fig. 1A). TEE further confirmed that although close to the superior vena cava, the mass did not quite obstruct venous drainage (Fig. 1B, [the mass did not show a good mobility on TEE] Video 1). Below the mass a 6 mm secundum atrial septal defect (ASD) was detected with left-to-right shunting. The size of atria and ventricle were in normal range and there was no valvular dysfunction or thrombus. The mass was not enhanced on computed tomography (CT) scan. There was high-intensity nodular and annular shadow around the inner wall and heterogeneous density inside (Fig. 2C). Magnetic resonance imaging (MRI) could not clearly show the interatrial septum (Fig. 2A and B). Deep vein thrombosis scan revealed no clots in lower extremity (external iliac and common femoral vein). After systemic evaluation a cardiopulmonary bypass cardiac surgery was performed to remove the interatrial septal mass. Intraoperation the mass was found to be wrapped in interatrial septum with a smooth and rough surface on the right and left atrial side respectively. We removed the whole mass intactly (Fig. 2D). After that, an artificial pericardium patch was sutured to close the defect and ASD. Postoperative course was uneventful. The ECG after surgery showed sinus rhythm and IBBB without atrioventricular block. The pathology of the mass proved to be organized thrombus with calcium deposition and fibrinoid necrosis. The patient was discharged with oral aspirin for 3 months and followed up as outpatient.

## 3 Discussion

ASA is a congenital heart disease. A typical ASA is not difficult to diagnose via TEE. However, in our case thrombus was formed and organized in the cavity of the ASA and makes it not distinguished from other common cardiac tumor. We compared the mass with some rare primary cardiac tumor to figure out the nature of the mass.

Myxoma is a relatively common primary cardiac tumor with a round shape and smooth, glistening surface. It usually locates in the left atrium and rarely in the ventricle. The typical characteristic of myxoma is that it is predunculated and oscillates periodically synchronized with heart. It could cause the stenosis of the mitral orifice and result in dyspnea. More seriously, the mucus fragment could lead to cerebral and peripheral embolism. The myxoma cells are stellate or fusiform with cytoplasm. The multi-nucleated myxoma cells may form rings, cords, and nests close to capillary.^[3]^ In our case, the solid mass does not coincide with most of the above typical features. Finally, the histopathology did not support the diagnose of myxoma.

Bronchogenic cyst is another possible cardiac tumor. Intracardiac bronchogenic cysts often attaches to the septum and protrudes into the cardiac cavities. The lumen of the cyst usually contains yellow, jelly-like fluid. Some cysts may cause dyspnea, chest pain, and arrhythmia according to the size, location, and compression of heart and vessels. The echo, MRI and CT could detect a cyst with a thin wall. Its typical histopathology is a cyst lining of ciliated columnar epithelium with interspersed goblet cells and surrounding fibrous connective tissue.^[4]^ In our case, the CT and MR both detected a heterogeneity inside the mass. Even we could not rule out the possibility of hemorrhage inside the bronchogenic cyst which caused the heterogeneity, the final histopathology which could not find any ciliated columnar epithelium still did not support the diagnose of bronchogenic cyst.

Cardiac blood cyst is a rare benign endocardiac tumor and frequent in pediatric age. It usually involves the valve apparatus. The patients could be asymptomatic or manifested with syncope, embolism, and valvular dysfunction, even sudden death.^[5]^ The cardiac blood cyst is thin-walled with an echo-free space in the lumen. The gross pathology is a cyst full of blood and sometimes with a supplying vessel. We could possibly rule out this diagnose before the surgery according to the above typical characteristics.

ASA is usually complicated with other congenital heart defects like patent foramen ovale and ASD with a reported prevalence of 32% and 3%, respectively.^[6]^ TEE is an effective examination to diagnose ASA, with a prevalence in TEE studies ranging from 2% to 10%.^[2]^ TEE is superior over TTE to diagnose ASA for a more consistent visualization of the interatrial septum. It was reported that 47% of ASA was missed with TTE.^[6]^ ASA is associated with atrial arrhythmia and maybe a possible source of arterial embolism.^[1]^ Three most popular and reasonable mechanisms underlying the phenomenon are paradoxical embolization through the patent foramen ovale or ASD, thrombus formation inside or around the ASA, and mitral valve prolapse which has been found to be frequently associated with ASA.^[7]^

With the advent of TTE and TEE, more interatrial septum aneurysms have been reported. Hanley had made a comprehensive summary about the characteristic echocardiography features of ASA with 80 patients.^[1]^ None of the 80 patients are detected with thrombus in ASA. However, it cannot be denied that thrombus could still form in ASA. Chammas had first reported a case of ASA with superimposed thrombus which caused multiple cerebral infarcts.^[8]^ This demonstrated that atrial septum aneurysm is a potential site of thrombus formation. Later Aryal also presented a case of spontaneous thrombus in the “teapot”-like ASA luckily without any embolism.^[9]^ In our case, the thrombus had formed for some time and gradually organized. It is not easy to make a clear diagnosis from the CT scan, MRI, or TEE as the thrombus organized in aneurysm made the ASA lose the distinguishable characteristic. The solid and heterogeneous mass with a broad base in the septum disguised itself as a tumor in the atrium. Fortunately, the patient had no embolism events in the formation of the thrombus in the ASA. However, a rough side of the thrombus in the left atrium may cause platelet activation and thrombsis. And the small ASD was also considered as a risk factor of paradoxical embolization. In this case, we think surgery is a reasonable and optimal option which can make a clear diagnosis with pathology and avoid the risk of embolism events in the future.

## 4 Conclusion

This case is a rare ASA fulfilled with organized thrombus which disguised itself as a cardiac tumor in the right atrium. The fact that it did not cause any pulmonary and systemic embolism is quite unusual and needs basic research to explore the mechanism of thrombus forming in the atrium.

## Author contributions

**Conceptualization:** Bo Zhang.

**Investigation:** Dehua Ma.

**Resources:** Chunguo Wang.

**Supervision:** Chengchu Zhu.

**Visualization:** Jiang Jin.

**Writing – original draft:** Xinxin Wang.

**Writing – review and editing:** Baofu Chen.

Xinxin Wang orcid: 0000-0003-1145-5361.

## Supplementary Material

Supplemental Digital Content
